# Change in Access to Prescribed Medication following an Episode of Deliberate Self-Poisoning: A Multilevel Approach

**DOI:** 10.1371/journal.pone.0098086

**Published:** 2014-05-22

**Authors:** Bergljot Gjelsvik, Fridtjof Heyerdahl, Daniel Lunn, Keith Hawton

**Affiliations:** 1 Department of Psychology University of Oslo, Oslo, Norway; 2 Department of Acute Medicine, Ullevaal, Oslo University Hospital, Oslo, Norway; 3 University of Oxford Department of Statistics, Oxford, United Kingdom; 4 Centre for Suicide Research, Oxford University Department of Psychiatry, Oxford, United Kingdom; Penn State College of Medicine, United States of America

## Abstract

**Objective:**

Patients with a history of deliberate self-poisoning (DSP) are prescribed a greater amount of medication than the general public. DSP is the most robust risk factor for repeat episodes of DSP and subsequent death by suicide, and one might therefore expect that access to prescribed medication would be reduced following an episode of DSP. However, it is unclear whether access to prescribed medication changes after an episode of DSP. The objectives of this study were to investigate changes in 1) overall, psychotropic, non-psychotropic and the psychotropic subgroup antidepressant prescribed medication availability in DSP patients following an episode of DSP, 2) prescribing of the medication ingested in the episode, and 3) potential effects of gender, age and repeater status on such change.

**Methods:**

The design was longitudinal. We included 171 patients admitted for DSP between January 2006 and March 2007. Data on patients' prescriptions prior to admission were retrieved from The Norwegian Prescription Database. The outcome measure was the difference between medication load in the year following compared to the year prior to the DSP episode.

**Results:**

There was a significant increase in total medication load following DSP, including both psychotropic and non-psychotropic medication. Antidepressant medication load remained stable. There was a tendency for access to drugs ingested in the episode to increase following the episode, albeit not significantly. Medication load increased with age across all medication groups irrespective of time period and gender.

**Conclusions:**

The findings show that physicians do not curb prescribing to patients who have recently deliberately self-poisoned. Moreover, they highlight the need for cautious and judicious prescribing for these patients, in combination with psychological and social interventions.

## Introduction

Approximately 90% of deliberate self-harm (DSH) episodes that present to hospitals involve deliberate self-poisoning (DSP) [1]. Monitoring and limiting access to medication therefore is a pivotal aspect of suicide prevention.

We have previously shown that patients with a history of DSP are prescribed an excessive amount of medication compared to the general public [2]. The majority of DSP patients use medication prescribed to them in their episodes [2], particularly those individuals who are severely depressed [3]. The much greater medication load found for DSP patients is likely to reflect a high degree of morbidity in this patient group [4]–[7]. In many cases, periods of high suicide risk are extremely brief [8], [9], thus access to medication may be a critical factor in turning compelling suicidal impulses to actual behaviour, including repeat episodes.

Little is known about what sort of treatment DSP patients receive following DSP episodes. To our knowledge no studies have explored whether and how prescribing to DSP patients changes following an episode of DSP. This is the case both for overall medication load, and in relation to specific drugs, such as antidepressants or drugs ingested in the episode. Our previous findings showed that it is the high medication load in general rather than the timing of single prescriptions in relation to time of episode that carries risk for deliberate self-poisoning [2]. Although medical conditions in most cases are unlikely to change abruptly following a DSP episode, the act itself may affect prescribing on an individual level either by serving as a marker of underlying problems not previously recognised by the prescriber(s), or by alerting prescribers to hitherto unacknowledged risks associated with on-going, long-term access to prescribed drugs. Moreover, the finding that the majority of DSP patients used medication prescribed to them in their DSP episodes [2] raises the question as to whether access to drugs ingested in the DSP episode changes after a DSP episode. To our knowledge no studies have explored whether and how prescribing to DSP patients changes following an episode of DSP. This is the case both for overall medication load and in relation to specific drugs, such as antidepressants or drugs ingested in the episode.

The objectives of this study were to investigate changes in 1) overall, psychotropic, non-psychotropic and antidepressant prescribed medication availability in DSP patients after compared with before an episode of DSP, 2) changes in prescribing of the medication ingested in the episode, and 3) potential effects of gender, age and repeater status on such change.

## Materials and Methods

### Design

Differences in medication load in the year following DSP episodes compared with the year beforehand were investigated in a longitudinal design comprising questionnaire as well as registry data for patients admitted to hospital for an episode of DSP.

### Data collection

DSP was defined as the intentional self-administration of more than the prescribed or recommended dose of any medication and with evidence that the act was intended to harm the patient though not necessarily to result in death [10]. The patients included in the study were individuals aged 18 years or older who had engaged in DSP and had presented to three major hospitals in Eastern Norway, Ullevål University Hospital, Oslo, Innlandet Hospital Trust, Gjøvik and Vestre Viken Hospital Trust, Bærum, between January 2006 and March 2007. These are all somatic hospitals, treating most cases of DSP. Patients treated without admission or referred directly from outpatient units, i.e., GP or psychiatric outpatient clinics, to psychiatric wards were not included in the study. Patients who were intellectually or developmentally disabled, psychotic or non-Norwegian speaking were excluded, as were patients admitted for accidental medication overdoses. The medical staff at the hospitals recruited the DSP cases to participate in the study while they were hospitalised (t1). Each patient completed a questionnaire, with the majority completing within the day following hospital presentation. Trained health personnel assisted the patients in completing the questionnaire.

### Measures

#### Prescription registry data

The outcome measure was difference between medication load in the year following compared to the year prior to the DSP episode. Data on patients' prescription records were retrieved from The Norwegian prescription database (NorPD) based on the patients' personal identification numbers.

By *medication load* we refer to the total amount of prescribed medication collected by an individual over a specified period of time. This may refer to medication load overall, or for subgroups, i.e., psychotropic and non-psychotropic medication. The measurement unit defined daily dose (DDD) is defined as the assumed average maintenance dose per day for a drug used for its main indication in adults (WHO Collaborating Centre for Drug Statistics Methodology). Drugs were described according to the Anatomical Therapeutic Chemical (ATC) classification system [11]. All collected medication was included in the analyses. Psychotropic drugs comprised antidepressants (ATC-code N06A), neuroleptics (ATC-code N05A), sedatives (ATC-codes N05B and N05C), anti-epileptics (ATC-code N03A), opioid analgesics (ATC-code N02A), non-opioid analgesics (ATC-codes N02B and M01A) and carisoprodol (ATC-code M03BA72). Albeit being classified as a muscle relaxant, carisoprodol was included in the psychotropic subgroup due to its frequent use in DSP [12].

#### Questionnaire data

In the current study, the following information from the patient self-report questionnaires was used: age, gender, repeater status at the time of the index episode (t1), i.e., whether the DSP episode that included them in the study was a repeat episode for that individual or a first-ever incident, and repeater status at t3, i.e., whether patients had made repeat episodes at 12 months follow-up. Patients also completed the Beck Depression Inventory Short Form (BDI-SF), a 13 items measure of level of depression [13].

#### Hospital data

Medication ingested in DSP episodes was identified by a physician who conducted a clinical evaluation of the medication involved based partly on clinical judgment and information from the patient, and partly on laboratory findings, (i.e., blood analyses) where necessary.

### Statistical analyses

Pre- and post-episode differences in collection of prescribed medication and potential effects of age, gender and repeater status were tested by fitting multilevel models using MLwiN version 2.24. The analysis took into account fact that the data are nested, i.e., dependency between various observations (i.e. collections) deriving from the same individual. The variance of the total DDD depends on the number of collections and this was taken into account by weighting according to that number. Model fit, i.e., normality of model residuals, was assessed using diagnostic plots supplemented by the Shapiro-Wilk normality test. All of the percentage increases quoted were obtained for the median patient age.

### Ethics

The study was approved by the East Regional Ethics Committee, the Privacy Ombudsman for Research as well as the Data Inspectorate. Written informed consent was obtained from all participating patients prior to inclusion in the study.

## Results

The whole sample was included in analyses regarding medication availability. Because only one hospital could provide data on drugs ingested in the DSP episode, only patients from this hospital were included in analyses regarding changes in collections of drugs ingested in the episode in the year following t1 compared to the year prior to the DSP episode.

All 286 eligible DSP patients treated for 378 DSP episodes during the study period were identified consecutively. Of the 286 patients, 177 (67.8% female and 32.2 male) consented to the researcher having access to information about prescriptions collected by them, yielding a 61.9% total response rate for registry data. For three patients, matching with registry data was not possible, for another three the method of self-harm did not involve DSP, yielding a sample of 171 patients for which registry data were obtained. In analyses regarding repeater status and depression level at t1, the number of patients included in the analyses were reduced to n = 128 due to missing values on these measures. Attrition analyses showed no gender differences between participating patients and those who declined to participate (p = 0.5), whereas older age predicted attrition (mean age in sample 38.6 years (Sd = 14.7) versus 43.7 years (Sd = 19.4) in non-participants, p = 0.02). We conducted a Wilcoxon signed rank test to investigate whether age and gender were confounded. Median age for males were significantly higher than for females (42 vs 35 yrs, respectively, p = 0.01).

Of the 117 eligible patients from the hospital where information was available on medication used for DSP, 85 patients consented to access to information about prescriptions dispensed to them, yielding a 72.6% response rate for registry data. After excluding one patient for whom matching with registry data was not possible there were 84 patients for whom registry data were obtained. The mean age of the patients was 37 years (SD = 14.8), which did not differ significantly from the mean age of participants from the two other hospitals (p = 0.2). The Shapiro Wilk normality test was used to confirm the validity of the normality assumptions for all the fitted models.

### 1. Total medication load from year before to year after the DSP episode

**Figure 1:**
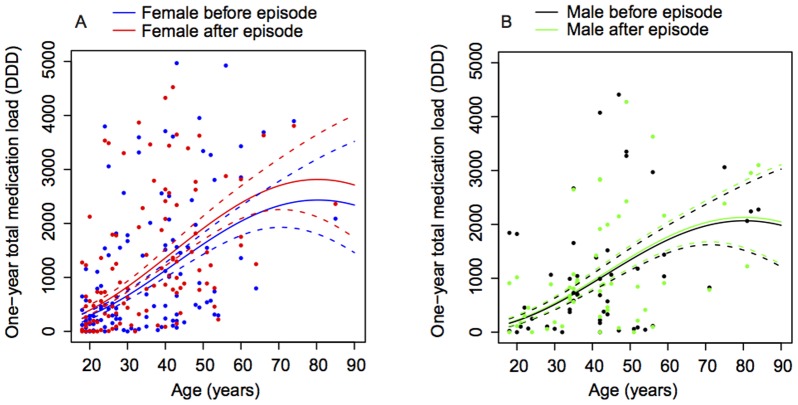
One year medication load by age and gender. Total medication load increased significantly in the year following the episode compared to beforehand (p<0.01). Figure 1A displays one year total medication load (DDD) for females by age before and after the episode, with the means (solid lines) and standard error envelopes (dashed lines). Females collected significantly more medication in the year after compared to the year prior to the episode (p<0.01), with the increase in DDD collected amounting to 21.2%. The pre-post difference for females gradually increased with age. There was no significant pre-post difference for males.

We took account of the repeated measures on each patient by fitting a multilevel model and included explanatory variables that may influence change in medication load from before to after a DSP episode (i.e., age, gender, depression at t1, repeater status at t1). Total medication load increased significantly in the year after the episode compared to beforehand (p<0.01). Females collected significantly more medication overall irrespective of age and time period (p = 0.01). Moreover, as shown in Fig. 1A, they also collected significantly more medication in the year after compared to the year prior to the episode (p<0.01), with the increase in DDD collected amounting to 21.2%. As shown in Fig. 1B, there was no significant pre-post difference for males. Total medication load increased with age irrespective of time period (p<0.01). This increase was not associated with depression level at t1 (p = 0.82). However, a significant interaction between gender and depression was found in that females collected more medication on the whole, irrespective of time period and age, compared to males with equal depression level. Repeater status at t1 had no bearing on the pre-post change in total medication load. Although the pre-post change was positive for all age groups, the pre-post difference for females increased gradually with age, as shown in Fig. 1A.

### 2. Psychotropic and non-psychotropic medication load

In order to delineate potential pre-post differences in psychotropic and non-psychotropic medication load, models were fitted separately for these two subgroups.

#### Psychotropic medication load

**Figure 2:**
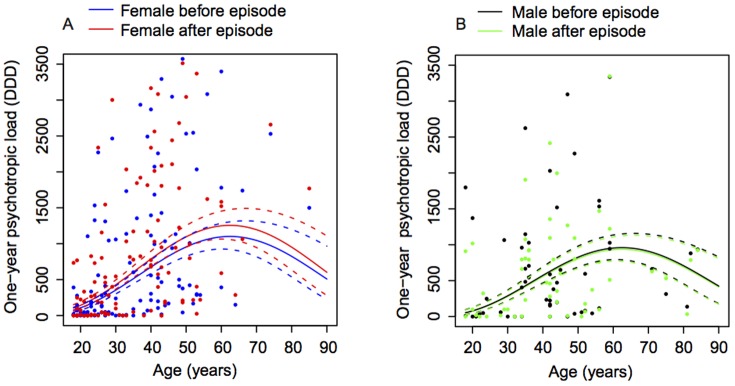
One year psychotropic load by age and gender. Figure 2A displays one year psychotropic medication load before and after the episode for females with the means (solid lines) and standard error envelopes (dashed lines). Psychotropic medication load increased significantly in the year after compared to the year prior to the episode (p = 0.04). Psychotropic medication load increased with age irrespective of time period and gender (p<0.01), and peaked in the 50–70 age bracket. There was no significant difference between males' and females' psychotropic medication load (p = 0.27).

As with total medication load, psychotropic medication load increased significantly in the year after compared to the year prior to the episode (p = 0.04). Whereas depression level at t1 was associated with the overall level of psychotropic medication (p<0.01), there was no effect of depression level at t1 on the pre-post change in psychotropic medication load (p = 0.15). Figure 2 shows psychotropic medication load year before and after the episode for females (2A) and males (2B), respectively, by age. Psychotropic medication load increased with age irrespective of time period and gender (p<0.01), and peaked in the 50–70 age bracket. There was no significant difference between males' and females' psychotropic medication load (p = 0.27).

#### Antidepressants medication load

We then investigated whether the psychotropic subgroup antidepressants showed a similar pattern to psychotropic drugs in general. There was no significant, demonstrable pre-post increase in antidepressant medication load (p = 0.42), and the total amount collected was not significantly different for males and females overall (p = 0.69). The pre-post increase in DDD collected was 9.7% for males and 9.4% for females, respectively, but this increase was not significant. There was no significant increase in any of the antidepressant subgroups tricyclic antidepressants, Selective Serotonin Reuptake Inhibitors (SSRIs), and others. Thus, there was no tendency for prescribers to replace the more toxic tricyclic subgroup with the less toxic SSRIs. However, antidepressant load increased with age irrespective of time period and gender (p<0.01).

#### Non-psychotropic medication load

**Figure 3:**
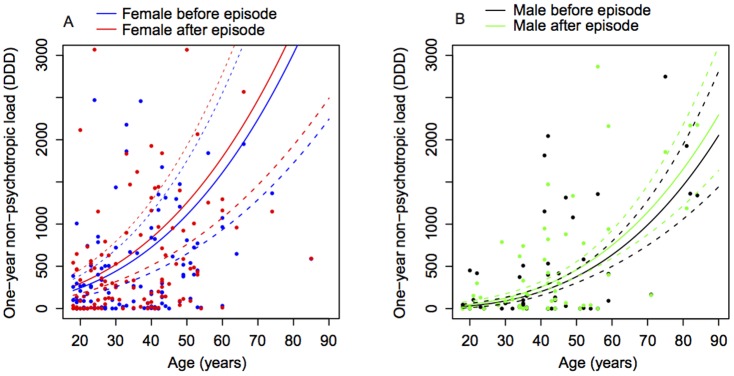
One year non-psychotropic load by age and gender. Figure 3A displays one year non-psychotropic medication load before and after the episode for females, and Figure 3B displays the corresponding information for males. Solid lines represent the means with dashed lines showing standard error envelopes. There was a significant increase in non-psychotropic medication load in the year after the episode (p<0.01) compared to the year prior to the DSP episode (a 24.3% increase in DDD collected), but this increase was not dependent on gender (p = 0.53). Non-psychotropic medication load increased with age (p<0.01), and females collected more non-psychotropic overall irrespective of age and time period (p = 0.01).

Figure 3 shows non-psychotropic medication load in the year before and year after the episode by age and gender (A: females, B: males). There was a significant increase in non-psychotropic medication load in the year after the episode (p<0.01) compared to the year prior to the DSP episode amounting to 24.3% increase in DDD collected). This increase was not dependent on gender (p = 0.53). Non-psychotropic medication load increased with age (p<0.01), and females collected more non-psychotropic overall irrespective of age and time period (p = 0.01). There was no significant interaction effect between gender and age.

### 3. Changes in medication load for drugs ingested in the episode

For the subgroup of patients who ingested prescribed drugs in the episode (n = 60), we investigated whether the medication load of these drugs changed from before to after the episode by fitting a multilevel model including gender, age and time period. There was a tendency for collections of medication ingested in the episode to increase following the episode, however this trend was not significant (p = 0.09). Females collected more drugs used in the DSP episode compared to men, irrespective of time period and age (p = 0.01), and as with the other categories of medication, this medication load increased with age irrespective of gender and time period (p<0.01). Neither depression level at t1 or repeater status at t1 was associated with changes in this medication load from the year before to the year after the episode.

### 4. Accumulation year following compared to the year prior to the DSP episode

**Figure 4:**
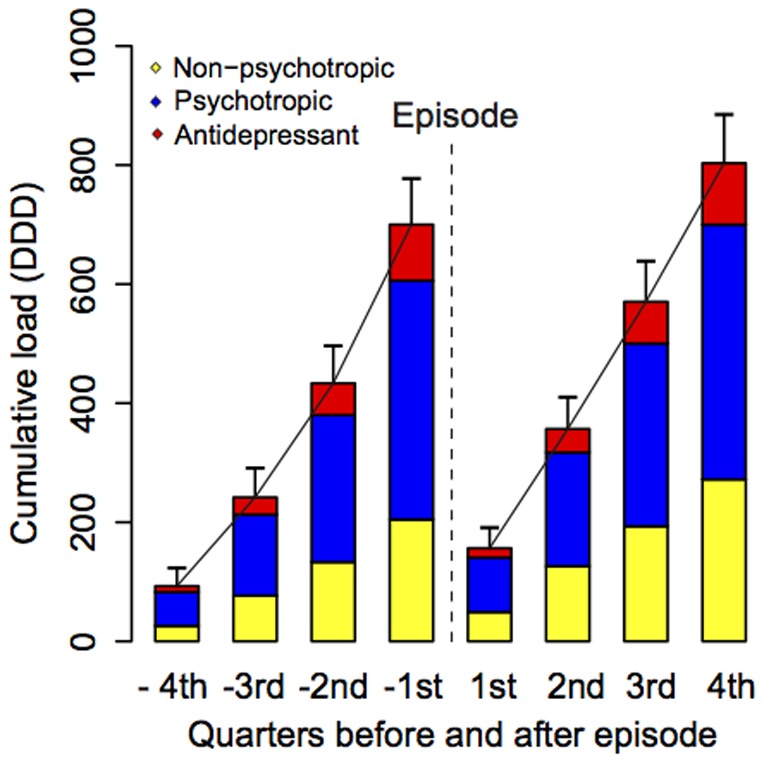
DDD per quarter year before and year after the episode by medication group, cumulating quarterly within each year. Comparison of year prior to and following the episode is potentially inaccurate as patients may be prescribed medication at different times in the two years, i.e., we may not be comparing the same time periods. We therefore investigated whether they were accumulating at the same rate in the two years. Figure 4 depicts the accumulated (total) DDD collected over the year prior to and the year following the episode, broken down into quarters and reported for non-psychotropic, psychotropic, and antidepressants within each quarter. The pace with which patients accumulated prescribed medication was steady over the two years, although the increase was faster over the first three months following the episode compared to the year preceding it.

Comparison of year prior to and following the episode is potentially inaccurate as patients may be prescribed medication at different times in the two years, i.e., we may not be comparing the same time periods. We therefore investigated whether they were accumulating at the same rate in the two years. Figure 4 depicts the accumulated (total) DDD collected over the year prior to and the year following the episode, broken down into quarters and reported for non-psychotropic, psychotropic, and antidepressants within each quarter. The pace with which patients accumulated prescribed medication was steady over the two years, although the increase was faster over the first three months following the episode compared to the corresponding period in the year preceding it.

## Discussion

We have previously reported the considerable medication load of DSP patients compared to the general public for the time leading up to the DSP episode [2]. Here we have investigated whether medication load changes in the year following the episode compared to the year beforehand. Our findings show that the amount of drugs collected increased significantly in the year following the episode compared to the year prior to the DSP episode.

### Pre-post change in total, psychotropic and non-psychotropic medication load

The pre-post increase for total medication load is perhaps not surprising given that the index DSP episode may have served to alert patients' general practitioners about hitherto unacknowledged mental health problems. However, the increase was not driven by an increase in psychotropic medication load alone: both types of medication increased. This is surprising, given that the level of physical morbidity in the sample is likely to remain relatively stable from one year to the next. Indeed, given that the majority of patients were in the younger age groups, it is unlikely that the increase can be accounted for by an increase in chronic physical disorders warranting medical treatment. However, we cannot rule out entirely the possibility that an increased morbidity in the sample around the period of the DSP episode, due to the one year increase in age from pre to post episode, have contributed to this increase.

It is possible that an adverse outcome such as a DSP episode prompted prescribers, in most cases, the general practitioner, to increase both frequency and quality of consultations. Increased and improved follow-up by prescribers may influence the total medication load in at least two ways: First, the attention of physicians to both physical and mental problems may be increased, with consequent increased prescribing including of new drugs. Second, previous studies have shown a strong association between suicidality and chronic physical conditions, and it has been suggested that this association is mediated in part by the hopelessness perceived in relation to the physical problems [14]. Hopelessness about physical conditions increases the risk of non-compliance, and polypharmacy, i.e., the use of several types of medication at the same time, has consistently been found to reduce overall compliance [15]. Thus the post-episodic rise in non-psychotropic medication load may in part reflect that prescriptions and collections become more aligned as a consequence of improved frequency and quality of health care follow-up. As such, the DSP episode may paradoxically serve to enhance the quality of medical health care provided.

The consistent increase in medication load with age is in keeping with the observed polypharmacy in the older population at large [15], [16] and the comparatively extensive use of opioids among the elderly [17], reflecting in part increased morbidity with age. Whilst no doubt motivated by and in many cases helpful in patients with high comorbidity, other non-medical factors contributing to polypharmacy, e.g., doctor-patient interaction, are less well understood. In patients vulnerable to DSP and thus to the toxic potential in polypharmacy these factors warrant particular attention.

The finding that females consistently collect more overall is in keeping with the tendency for females to collect more prescriptions overall in the population at large. Interestingly, however, there was no marked gender difference in psychotropic medication load. This is contrary to what has been found in a recent population-based study of psychotropic drug use [18]. One possible reason for this discrepancy is that all patients in our study had engaged in suicidal behaviour and thus potentially represent a subgroup within which gender differences are less pronounced than in the population at large. This warrants further study.

The pre-post increase in psychotropic medication is less surprising. Not only may the episode have served as an indicator of underlying mental illness not previously recognised – the episode may also have served as an indicator that current medical treatment for mental problems warranted adjustment, i.e., the pre-post psychotropic increase may reflect a recognition of under-prescribing of psychotropic medication. Due to the strong association between suicidal behaviour and depression, this would have been likely to be seen in the antidepressant subgroup. However, the antidepressant medication load was relatively stable, possibly reflecting that these patients were already prescribed antidepressants. It is likely that some of the mechanisms suggested to explain the pre-post increase in non-psychotropic medication, such as increased quality and frequency of follow-up; or increased compliance, are at play for psychotropic prescribing as well.

### Strengths and limitations of this study

A main strength of the present study is that it is based on a precise measure of access to prescription-based medication at an individual level. In contrast to other studies the data have been analysed longitudinally, thus enabling us to investigate the changes in access to prescribed medication following an episode of DSP. Moreover, the application of multilevel modelling ensures that the nested nature of the data was taken into account. Furthermore, medication ingested at the time of the DSP episode was evaluated consecutively by a physician. Finally, rather than using data of what was prescribed, we have looked at what types of medication were actually collected and hence available. However, the study has several limitations. First, the current data do not exclude the possibility that the pre-post increase observed reflects a time trend due to factors other than the DSP episode. To ascertain this would require longer periods of observation. Whilst outside the scope of this study this is undoubtedly a topic for further research. Secondly, while it includes patients presenting to three hospitals, analyses of pre-post differences in episodic medication load are based on patients from one hospital. Thirdly, analyses of prescriptions taken in overdose do not include OTC medication. Fourthly, selection bias might limit the generalizability of the findings. Because only patients who were admitted to hospital for their DSP were included, the findings cannot be generalized to less severe cases of DSP that are not admitted, which are relatively few, nor other types of deliberate self-harm. Moreover, the non-significant finding for changes in medication load for drugs ingested in the episode may reflect an insufficient sample size, and warrant further study. Finally, the higher mean age among persons declining to participate might indicate a more ‘chronic’ sample. However, our data did not suggest this.

## Conclusions

The increased prescribing of drugs in the year following the DSP episode shows that physicians do not curb prescribing to patients who have recently deliberately self-poisoned. Given the well-founded risks related to availability of means of suicide [19], findings of this study highlight the need for cautious and judicious prescribing for these patients, involving prescribing lowest effective dose on a more frequent basis, in combination with psychological and social interventions [20].
